# Diagnostic Accuracy and Reliability of Noncontrast Computed Tomography Markers for Acute Hematoma Expansion among Radiologists

**DOI:** 10.3390/tomography8060242

**Published:** 2022-12-09

**Authors:** Hawra Almubarak, Sarah Elsayed, Federico Mazzacane, Frieder Schlunk, Haoyin Cao, Ly Huong Vu, Estelle Vogt, Andrea Dell Orco, Dmitriy Desser, Maik F. H. Böhmer, Burak Han Akkurt, Peter B. Sporns, Tobias Penzkofer, Uta Hanning, Andrea Morotti, Jawed Nawabi

**Affiliations:** 1 Department of Radiology (CCM), Berlin Institute of Health, Charité—Universitätsmedizin Berlin, Campus Mitte, Humboldt-Universität zu Berlin, Freie Universität Berlin, 10117 Berlin, Germany; 2 Department of Diagnostic and Interventional Neuroradiology, University Medical Center Hamburg Eppendorf, 20251 Hamburg, Germany; 3 Department of Brain and Behavioral Sciences, University of Pavia, 27100 Pavia, Italy; 4 Malattie Cerebrovascolari e Stroke Unit, IRCCS Fondazione Mondino, 27100 Pavia, Italy; 5 Department of Neuroradiology (CCM), Berlin Institute of Health, Charité—Universitätsmedizin Berlin, Campus Mitte, Humboldt-Universität zu Berlin, Freie Universität Berlin, 10117 Berlin, Germany; 6 Berlin Institute of Health (BIH), BIH Biomedical Innovation Academy, 10117 Berlin, Germany; 7 Department of Radiology, University Hospital Muenster, 48149 Muenster, Germany; 8 Department of Neuroradiology, Clinic for Radiology and Nuclear Medicine, University Hospital Basel, 4031 Basel, Switzerland; 9 Department of Radiology (CCM), Berlin Institute of Health, Charité—Universitätsmedizin Berlin, Campus Virchow Klinikum, Humboldt-Universität zu Berlin, Freie Universität Berlin, 13353 Berlin, Germany; 10 Neurology Unit, Department of Neurological Sciences and Vision, ASST-Spedali Civili, 25123 Brescia, Italy

**Keywords:** intracerebral hemorrhage, NCCT marker, reliability, level of experience

## Abstract

Background: Noncontrast Computed Tomography (NCCT) features are promising markers for acute hematoma expansion (HE) in patients with intracerebral hemorrhage (ICH). It remains unclear whether accurate identification of these markers is also reliable in raters with different levels of experience. Methods: Patients with acute spontaneous ICH admitted at four tertiary centers in Germany and Italy were retrospectively included from January 2017 to June 2020. In total, nine NCCT markers were rated by one radiology resident, one radiology fellow, and one neuroradiology fellow with different levels experience in ICH imaging. Interrater reliabilities of the resident and radiology fellow were evaluated by calculated Cohen’s kappa (κ) statistics in reference to the neuroradiology fellow who was referred as the gold standard. Gold-standard ratings were evaluated by calculated interrater κ statistics. Global interrater reliabilities were evaluated by calculated Fleiss kappa statistics across all three readers. A comparison of receiver operating characteristics (ROCs) was used to evaluate differences in the diagnostic accuracy for predicting acute hematoma expansion (HE) among the raters. Results: Substantial-to-almost-perfect interrater concordance was found for the resident with interrater Cohen’s kappa from 0.70 (95% CI 0.65–0.81) to 0.96 (95% CI 0.94–0.98). The interrater Cohen’s kappa for the radiology fellow was moderate to almost perfect and ranged from 0.58 (95% CI 0.52–0.65) to 94 (95% CI 92–0.97). The intrarater gold-standard Cohen’s kappa was almost perfect and ranged from 0.79 (95% CI 0.78–0.90) to 0.98 (95% CI 0.78–0.90). The global interrater Fleiss kappa ranged from 0.62 (95%CI 0.57–0.66) to 0.93 (95%CI 0.89–0.97). The diagnostic accuracy for the prediction of acute hematoma expansion (HE) was different for the island sign and fluid sign, with *p*-values < 0.05. Conclusion: The NCCT markers had a substantial-to-almost-perfect interrater agreement among raters with different levels of experience. Differences in the diagnostic accuracy for the prediction of acute HE were found in two out of nine NCCT markers. The study highlights the promising utility of NCCT markers for acute HE prediction.

## 1. Introduction

Intracerebral hemorrhage (ICH) is the deadliest type of stroke, with a mortality rate of 40% in the first month and severe morbidity in most survivors [1]. Hematoma size is the strongest predictor of unfavorable outcome, and up to half of patients experience early hematoma expansion (HE) [2]. Hematoma expansion (HE) is potentially preventable, and therefore the rapid identification of patients at high risk of active bleeding is crucial for the development of anti-expansion therapies [3]. Noncontrast Computed Tomography (NCCT) features have emerged as promising markers identifying acute HE upon admission imaging [4]. Consensus criteria proposed by the international NCCT study group have aimed at paving the way toward a standardization of NCCT marker nomenclature [5]. Further clinical studies have reported high levels of reliability for these markers [6,7]. However, these markers were analyzed by raters with a strong expertise in ICH neuroimaging. It remains unclear whether rapid and accurate identification of these NCCT markers also performs well among raters with different levels of experience in ICH neuroimaging. We hypothesized firstly that NCCT markers rated by different experienced raters would be variable and secondly that their experience would have an influence on the predictive effectiveness. To test and evaluate these two hypotheses, ratings of one radiology resident, one radiology fellow, and one neuroradiology fellow with different levels of experience in ICH imaging were evaluated. Differences in their predictive value for outcome prediction arising from potential discordant ratings were analyzed.

## 2. Methods

### 2.1. Study Population

We retrospectively selected ICH patients admitted at four tertiary stroke centers in Germany and Italy (Charité University Hospital, Berlin, Germany (2015–2019); University Medical Center Hamburg-Eppendorf (2015–2019), Germany; University Hospital Muenster (2011–2015), Germany; and IRCCS Mondino Foundation, Pavia, Italy (2017–2019)). Patients were selected according to the following inclusion criteria: primary, spontaneous ICH, age > 18 years, admission non-contrast computed tomography (NCCT) images acquired within 24 h from onset/last seen well (LSW). Patients with secondary ICH were excluded from the analysis. Clinical data obtained from medical records included age, sex, history of hypertension and diabetes mellitus, systolic blood pressure, anticoagulation and antiplatelet treatment, Glasgow Come Scale (GCS) at admission, time from symptom onset/LSW to imaging, and modified Rankin Scale (mRS) at 90 days.

### 2.2. Image Analysis

NCCT images were acquired based on local CT protocols at each participating site. Imaging data were retrieved in Digital Imaging and Communications in Medicine (DICOM) format from the local picture archiving and communication system (PACS) servers and anonymized in compliance with the local guidelines. DICOM data were transformed into a Neuroimaging Informatics Technology Initiative (NifTI) format for further image analysis. Images were analyzed for the presence of IVH (intraventricular hemorrhage) and ICH location. Supratentorial bleedings in cortical and subcortical location were classified as lobar whether or not hemorrhages involving the thalamus, basal ganglia, internal capsule, and deep periventricular white matter were classified as deep [8]. Brainstem and cerebellar bleedings were classified as infratentorial [9]. Volume quantifications of ICH and IVH were performed on NCCT images with semimanual planimetric measurements. The ROI histogram for ICH and IVH segmentation was sampled between 20 and 80 Hounsfield units (HU) to exclude voxels that likely belonged to cerebrospinal fluid or calcification. Regions of interest (ROIs) were delineated by using Analyze 11.0 Software and ITK-SNAP 3.8.0 Software [10,11]. HE and revised HE were both calculated as previously reported [12]. All NCCT markers were rated on axial NCCT images for the following nine markers, namely (1) irregular (IRR) shape, (2) satellite sign, (3) island sign, (4) heterogenous (HET) density, (5) swirl sign, (6) black hole sign, (7) blend sign, (8) fluid sign, and (9) hypodensities, using definitions proposed by the International NCCT Study Group (Supplementary Materials Table S1) [4,5,13,14,15,16,17,18,19,20,21]. NCCT markers were reviewed on all images by three raters: A neuroradiology fellow (J.N., with 5 years of experience in stroke imaging), one resident (H.A., with 4 years of stroke imaging experience and specific experience in the utility of NCCT markers in clinical research), and one radiology fellow (S.E., with formal stroke imaging training during the residency program including the utility of NCCT markers). Images for the second reading of the neuroradiology fellow were presented in a random order three months later to minimize the recall of the images. All readers independently reviewed images in a random order, blinded to all demographic and outcome data and were not directly involved in the clinical care of the enrolled patients.

### 2.3. Statistical Analysis

Data were tested for normality and homogeneity of variance by using histogram plots and the Shapiro–Wilk test. Descriptive statistics are presented as counts (percentages, %) for categorical variables and compared with χ2 test, mean (standard deviation, SD) for continuous normally distributed variables, and medians (interquartile range, IQR) for non-normal continuous variables and compared with the Mann–Whitney test, respectively. Interrater agreements were calculated and expressed as Cohen’s κ statistic with stratified kappa with 95% upper and lower confidence intervals (CIs) from pairs of two readers (neuroradiology fellow and radiology resident; neuroradiology fellow and radiology fellow) [22,23]. Intrarater agreement for the neuroradiology fellow’s readings of NCCT markers was calculated and expressed as Cohen’s κ statistic with stratified kappa and 95% upper and lower CI [24]. Global interrater agreement was calculated and expressed as Fleiss κ statistic with stratified kappa with 95% upper and lower CI across all three readers (radiology resident; radiology fellow and neuroradiology fellow). The strength of agreement was defined as “poor”, with a κ < 0.00; “light”, with a κ of 0.00–0.20; “fair”, with a κ of 0.21–0.40; “moderate”, with a κ of 0.41–0.60; “substantial”, with a κ of 0.61–0.80; and “almost perfect”, with a κ of 0.81–1.00. The diagnostic performance of the NCCT markers to predict acute HE was assessed for each rater independently by conducting a Receiver Operating Curve (ROC) analysis with increasing discrimination thresholds [25]. A pairwise ROC comparison was performed by assessing differences in the areas under the empirical ROC curves (AUC) and statistically evaluated with a de Long et al. test [26]. A statistically significant difference was accepted at a *p*-value of less than 0.05.

## 3. Results

**Figure 1:**
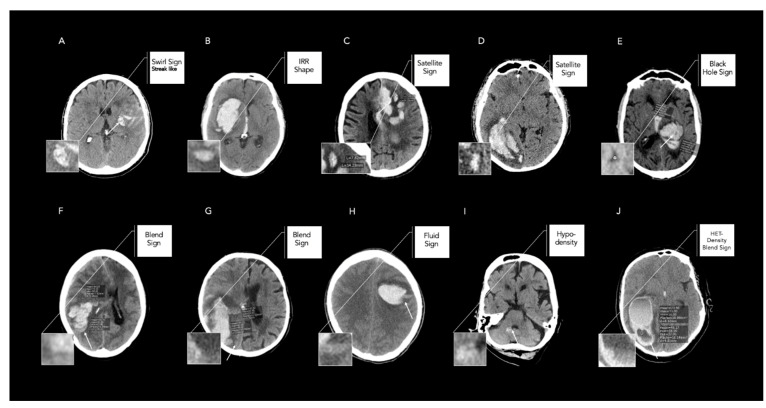
Illustrative examples of false-positive and -negative rated Noncontrast Computed Tomography (NCCT) markers between different raters. Legend: Illustrative examples of interpretation of Noncontrast Computed Tomography markers with maximal discordances between one radiology resident, one radiology fellow, and one neuroradiology fellow. (**A**) Streak-like swirl sign was rated false negative by the resident. (**B**) IRR shape rated false negative as Barras II versus qualifying as Barras III by the neuroradiology fellow. (**C**) Satellite sign rated false negative, with a diameter greater than 10 mm, as shown by the neuroradiology fellow’s measurements. (**D**) Satellite sign rated false positive, as the small hematoma shows signs of connection with the main hematoma according to the fellow. (**E**) Black-hole sign rated false positive, with encapsulated hypodensity but density differences less than 28 HU, as shown by the neuroradiology fellow’s measurements. (**F**,**G**) Blend sign rated false positive, with well-defined margins of density changes within the hematoma but density differences less than 18 HU, as indicated by the neuroradiology fellow’s measurements. (**H**) Fluid sign rated false positive with a discrete straight line above the hyperattenuating area which is not clearly separated by a hypoattenuating area of hemorrhage or edema. (**I**) hypodensity rated false positive as no strict encapsulation was defined by the neuroradiology fellow. (**J**) HET density rated correctly by two raters. Fluid sign was rated by neuroradiology fellow. In contrast, blend sign was rated false positive, with density measurements less than 18 HU, by the resident.

A total of 735 patients with acute ICH were included with a median age of 73 years (IQR 62–80) and 56.9% female patients. Further details are presented in Table 1. The distribution of the NCCT markers varied across all ratings, as shown in Table 2. The interrater reliabilities between the radiology fellow and neuroradiology fellow demonstrated overall substantial-to-almost-perfect levels of agreement, as shown in Table 3. The levels of agreement between the radiology resident and the neuroradiology fellow were comparatively moderate to almost perfect, yielding the lowest levels of agreement for the swirl sign, with a Cohen’s κ of 0.58 (95% CI 0.52–0.65), as shown in Table 3. Illustrative examples of maximal discordances between the raters are shown in Figure 1. The global interrater agreement was moderate to almost perfect, with the Fleiss κ ranging from 0.62 (95% CI 0.57–0.66) for the swirl sign to 0.93 (95% CI 0.89–0.97) for the fluid sign, as shown in Table 3. The quality of gold-standard ratings was assessed with substantial-to-almost-perfect intrarater agreements, as shown in Table 4. The AUC comparisons between the raters for predicting acute HE were significantly different for the island and fluid sign (Supplementary Materials Table S2).

## 4. Discussion

In this study, we aimed to determine the link between the level of experience in raters and the reliability of the assessment of NCCT markers. Our previous results demonstrated good-to-excellent levels of inter- and intrarater reliability and contribute to the results presented by Dowlatshahi et al. [6,7]. However, the different levels of experience amongst raters with a radiological background may result in significant interobserver variability and differences in the diagnostic accuracy for predicting acute HE. Therefore, the results of our analysis highlight several novel important findings. Firstly, the reliability of NCCT markers varied among raters with different levels of experience. Nevertheless, eight out of nine NCCT markers showed substantial-to-almost-perfect agreement, whereas a moderate agreement was only found for the swirl sign. The illustrative examples shown in Figure 1 demonstrate that especially very nuanced density changes, such as the streak-like morphology of the swirl sign (Figure 1A) or scattered, primarily satellite-suggestive hematoma with yet subtle connections to the main hematoma (Figure 1D), were difficult to identify. Moreover, the strict encapsulation of the hypodense area within the hematoma (hypodensities) was false positively rated, especially in cases of small hematomas with very nuanced NCCT feature attributes, as shown in Figure 1I. Semiquantitative measurements were error-prone for calculating the correct diameter of the hemorrhage for assessing the satellite sign (Figure 1C,D) and density differences for the black hole sign or blend sign (Figure 1E–G). In line with this, raters from different clinical backgrounds also tended to obtain higher proportions of positive ratings of NCCT markers according to a recently published study [27]. Variabilities in the ratings of the IRR shape, heterogenous density, and island sign may be further influenced by differences in the slice position of the region of interest (ROI), which should be placed on the axial slice with the largest cross-sectional area of the hematoma (Figure 1B,J) [5]. This is of clinical importance, as measurement error may potentially obscure the true predictive effects. Evaluated AUC differences for acute HE prediction were minor and found in only two out of nine NCCT markers. Our study had some limitations. Firstly, our study offered only limited conclusions to whether variances of agreements were attributed to the level of experience, as this would require a larger sample size within each category of experience included. Furthermore, given that the imaging analysis in an emergency setting is often much more rushed than in an elective case, accounting for the reading time may have resulted in additional variability of the readings. Secondly, our findings were derived from a retrospective analysis and require prospective confirmation. Finally, the imaging protocol was not standardized across participating sites. Nevertheless, there is no evidence that the NCCT acquisition technique influences NCCT markers’ detection [5].

## 5. Conclusions

In conclusion, the reliability for NCCT markers varied among raters of different levels of experience with a radiological background. Overall, the interrater reliability was moderate to almost perfect. Differences in the predictive performance were minor and found in two out of nine NCCT markers. Our findings highlight the promising utility of NCCT markers for the prediction of acute HE. Future studies may include a larger sample size of raters with different clinical backgrounds towards addressing the potential impact of the level on experience on the reliability of the proposed markers.

## Figures and Tables

**Table 1:** Baseline demographics, as well as clinical and radiological characteristics in patients with and without intraventricular hemorrhage growth (IVH growth), in patients with intracerebral hemorrhage.

Baseline Characteristics	All (*n* = 735)
Age [years], median (IQR)	73 (62–80)
Female, *n* (%)	410 (56.9)
Systolic RR [mmHg], median (IQR)	165 (145–195)
Hypertension, *n* (%)	581 (80.7)
Diabetes mellitus, *n* (%)	121 (16.6)
Anticoagulation Treatment, *n* (%)	201 (27.9)
Antiplatelet Treatment, *n* (%)	324 (45.0)
GCS admission, median (IQR)	13 (9–15)
Δ symptom onset to imaging [h], median (IQR)	6.23 (1.65–17.55)
Craniotomy, *n* (%)	116 (16.11)
**Hemorrhage Characteristics**	
ICH Volume on admission [mL], mean (SD)	44.56 (5.99)
ICH Volume on follow-up [mL], mean (SD)	60.86 (16.42)
Intraventricular hemorrhage on admission, *n* (%)	327 (45.42)
Intraventricular hemorrhage on follow-up, *n* (%)	363 (50.4)
HE [>6 mL; >33%], *n* (%)	150 (20.8)
revised HE [HE; IVH growth], *n* (%)	271 (37.7)
**Location characteristics**	
Supratentorial, *n* (%)	615 (85.4)
Lobar, *n* (%)	309 (43)
Basal ganglia, *n* (%)	306 (42.6)
Thalamic, *n* (%)	36 (5.7)
Brainstem/pons, *n* (%)	69 (9.6)
Cerebellar, *n* (%)	35 (4.9)
**Clinical Outcome**	
mRS 0–3, *n* (%)	190 (26.39)
mRS 4–6, *n* (%)	530 (73.61)
mRS 6 (mortality), *n* (%)	188 (26.10)

Legend: HE, hematoma expansion; ICH, intracerebral hemorrhage; IQR, interquartile range; IVH, intraventricular hemorrhage; GCS, Glasgow Come Scale; mRS, modified Rankin Scale; RR, arterial blood pressure.

**Table 2:** Distribution of Noncontrast Computed Tomography Signs (NCCT Signs) in patients with acute intracerebral hemorrhage.

NCCT Marker (*n* = 735)	Neuroradiology Fellow	Radiology Resident	Radiology Fellow	*p*-Value
**Shape Markers**				
IRR Shape, *n* (%)	467 (63.54)	466 (63.40)	469 (63.81)	<0.001
Satellite Sign, *n* (%)	300 (40.82)	285 (38.78)	311 (42.31)	<0.001
Island Sign, *n* (%)	347 (47.21)	328 (44.63)	298 (40.54)	<0.001
**Density Markers**				
HET Density, *n* (%)	191 (25.99)	162 (22.05)	177 (24.08)	<0.001
Swirl Sign, *n* (%)	488 (66.39)	475 (64.63)	529 (71.97)	<0.001
Black Hole Sign, *n* (%)	192 (26.12)	175 (23.81)	148 (20.14)	<0.001
Blend Sign, *n* (%)	81 (11.02)	79 (10.75)	75 (10.220)	<0.001
Fluid Sign, *n* (%)	49 (6.67)	46 (6.26)	43 (5.85)	<0.001
Hypodensities, *n* (%)	325 (44.22)	356 (48.44)	296 (36.60)	<0.001

Legend: Distribution of nine Noncontrast Computed Tomography markers rated between a radiology resident, radiology fellow, and neuroradiology fellow. HET density indicated heterogeneous density; IRR shape, irregular shape.

**Table 3:** Agreement of Noncontrast Computed Tomography markers across raters with different levels of experience stratified with Cohen’s kappa and the Fleiss kappa.

NCCT Marker	Rater	Cohen’s Kappa (95% CI)	Rater	Fleiss Kappa (95% CI)
**Shape Markers**				
IRR Shape	Rad Resident and Neurorad Fellow	0.88 (0.85–0.92)	Rad Resident, Rad Fellow, and Neurorad Fellow	0.90 (0.86–0.94)
	Rad Fellow and Neurorad Fellow	0.94 (0.92–0.97)		
Satellite Sign	Rad Resident and Neurorad Fellow	0.94 (0.91–0.96)	Rad Resident, Rad Fellow, and Neurorad Fellow	0.80 (0.76–0.84)
	Rad Fellow and Neurorad Fellow	0.86 (0.78–0.93)		
Island Sign	Rad Resident and Neurorad Fellow	0.95 (0.92–0.97)	Rad Resident, Rad Fellow, and Neurorad Fellow	0.86 (0.82–0.91)
	Rad Fellow and Neurorad Fellow	0.78 (0.71–0.84)		
**Density Markers**				
HET Density	Rad Resident and Neurorad Fellow	0.85 (0.80–0.89)	Rad Resident, Rad Fellow, and Neurorad Fellow	0.86 (0.82–0.9)
	Rad Fellow and Neurorad Fellow	0.94 (0.91–0.97)		
Swirl Sign	Rad Resident and Neurorad Fellow	0.96 (0.94–0.98)	Rad Resident, Rad Fellow, and Neurorad Fellow	0.62 (0.57–0.66)
	Rad Fellow and Neurorad Fellow	0.58 (0.52–0.65)		
Black Hole Sign	Rad Resident and Neurorad Fellow	0.94 (0.91–0.97)	Rad Resident, Rad Fellow, and Neurorad Fellow	0.79 (0.75–0.84)
	Rad Fellow and Neurorad Fellow	0.75 (0.66–0.84)		
Blend Sign	Rad Resident and Neurorad Fellow	0.70 (0.65–0.81)	Rad Resident, Rad Fellow, and Neurorad Fellow	0.79 (0.75–0.83)
	Rad Fellow and Neurorad Fellow	0.77 (0.67–0.88)		
Fluid Sign	Rad Resident and Neurorad Fellow	0.96 (0.91–1.00)	Rad Resident, Rad Fellow, and Neurorad Fellow	0.93 (0.89–0.97)
	Rad Fellow and Neurorad Fellow	0.92 (0.86–0.98)		
Hypodensities	Rad Resident and Neurorad Fellow	0.84 (0.80–0.88)	Rad Resident, Rad Fellow, and Neurorad Fellow	0.83 (0.79–0.87)
	Rad Fellow and Neurorad Fellow	0.83 (0.78–0.87)		

Legend: Interrater agreement for nine different Noncontrast Computed Tomography (NCCT) markers with stratified Cohen’s kappa and the Fleiss kappa across one radiology resident, one radiology fellow, and one neuroradiology fellow stratified across one reading. Neuro Fellow, neuroradiology fellow; Rad Fellow, radiology fellow.

**Table 4:** Agreement of Noncontrast Computed Tomography markers across one gold rater stratified with Cohen’s kappa.

NCCT Marker	Intrarater	Cohen’s Kappa (95% CI)	*p*-Value
**Shape Markers**			
IRR Shape	Neurorad Fellow	0.87 (0.83–0.91)	<0.001
Satellite Sign	Neurorad Fellow	0.93 (0.88–0.97)	<0.001
Island Sign	Neurorad Fellow	0.95 (0.90–1.00)	<0.001
**Density Markers**			
HET Density	Neurorad Fellow	0.79 (0.73–0.84)	<0.001
Swirl Sign	Neurorad Fellow	0.94 (0.91–0.98)	<0.001
Black Hole Sign	Neurorad Fellow	0.98 (0.95–1.00)	<0.001
Blend Sign	Neurorad Fellow	0.96 (0.90–1.00)	<0.001
Fluid Sign	Neurorad Fellow	0.95 (0.90–1.00)	<0.001
Hypodensities	Neurorad Fellow	0.81(0.78–0.86)	<0.001

Legend: Intrarater agreement for nine different Noncontrast Computed Tomography (NCCT) markers with stratified Cohen’s kappa across one neuroradiology fellow stratified across two reading. Neurorad Fellow, neuroradiology fellow.

## Data Availability

The datasets that support the findings of our study are available upon reasonable request from the corresponding author; however, prior approval of proposals may be apply for from our institution’s data security management, and a signed data-sharing agreement will then be approved.

## Supplementary Materials

The following supporting information can be downloaded at https://www.mdpi.com/article/10.3390/tomography8060242/s1. Table S1: Definitions for Noncontrast Computed Tomography Markers. Table S2: Receiver operative characteristic analysis for Noncontrast Computed Tomography Markers in the prognosis of acute hematoma expansion between different raters.
