# Supplementary material for: Diagnostic Accuracy and Reliability of Noncontrast Computed Tomography Markers for Acute Hematoma Expansion among Radiologists
Source: Tomography. 2022 Dec 9;8(6):2893–901. doi: 10.3390/tomography8060242 (PMC9785236; doi:10.3390/tomography8060242)
Supplement: Supplementary file 1 [file tomography-08-00242-s001.zip › tomography-2016155-supplementary.pdf]

Supplementary Material

Table S1: Definitions for Noncontrast Computed Tomography Markers

NCCT marker	Definition
IRR shape	IRR shape was defined as foci of hematoma margin irregularities in the largest hematoma region according to the Barras shape scale I-V. The presence of IRR shape was defined after dichotomization with a Barras scale of ≥ III.
Satellite sign	Satellite sign was defined as a hematoma separate from the main hematoma (1-20 mm distance) with a maximum diameter of 10 mm.
Island sign	The island sign consisted of at least three scattered small hematomas all separate from the main hematoma or at least four small hematomas some or all of which may connect with the main hematoma.
HET density	HET density was evaluated as foci of hypoattenuation within the largest hematoma compared to the brain parenchyma according to the Barras density scale I-V. The presence of HET density was defined after dichotomization with a Barras scale of ≥ III.
Swirl sign	The swirl sign was defined as a region of hypo- or isoattenuation compared with the brain parenchyma. The region may be rounded, streak-like or irregular and does not require a strict encapsulation within the hematoma.
Black hole sign	The black hole sign consisted of a relatively hypodense area which is encapsulated within a hyperdense area and which is not connected with the adjacent brain tissue. The relatively hypodense area has an identifiable border and a difference of at least 28 HU between the two density regions.
Blend sign	Blend sign was defined as a hypoattenuating area adjacent to a hyperattenuating area of the hematoma, with a clear separation between them at a density difference of at least 18 Hounsfield Units (HU).
Fluid sign	Fluid sign referred similarly to the presence of one distinct hypoattenuating area above and one hyperattenuating area below a discrete straight line of separation, yet irrespective of its density measurements.
Hypodensities	The imaging sign hypodensities was defined as any hypodense region strictly encapsulated within the hemorrhage with any shape, size, and density which does not require a density measurement.

Legend: Definitions on Noncontrast Computed Tomography markers in patients with acute spontaneous intracerebral hemorrhage. HET density indicated heterogeneous density; IRR shape, irregular shape; NCCT, noncontrast Computed Tomography.

Table S2: Receiver operative characteristic analysis for Noncontrast Computed Tomography Markers in the prognosis of acute hematoma expansion between different raters.

IRR Shape	AUC	95%CI	SE	
Neuroradiology Fellow	0.585	0.548-0.621	0.018	
Resident	0.589	0.549-0.622	0.018	
Radiology Fellow	0.586	0.552-0.625	0.018	
AUC comparison	Δ AUC	95%CI	SE	*P*-value*
Neuro Fellow ~ Resident	0.004	-0.012-0.02	0.008	0.616
Resident ~ Rad Fellow	0.003	-0.013-0.02	0.009	0.701
Neuro Fellow ~ Rad Fellow	0.001	-0.01-0.012	0.005	0.894

Satellite Sign	AUC	95%CI	SE	
Neuroradiology Fellow	0.528	0.492-0.565	0.019	
Resident	0.521	0.485-0.558	0.019	
Radiology Fellow	0.521	0.484-0.558	0.013	
AUC comparison	Δ AUC	95%CI	SE	*P*-value*
Neuro Fellow ~ Resident	0.007	-0.007-0.021	0.007	0.336
Resident ~ Rad Fellow	0.000	-0.037-0.0370	0.012	0.994
Neuro Fellow ~ Rad Fellow	0.007	-0.029-0.044	0.019	0.707

Island Sign	AUC	95%CI	SE	
Neuroradiology Fellow	0.566	0.507-0.581	0.019	
Resident	0.530	0.493-0.566	0.019	
Radiology Fellow	0.523	0.472-0.573	0.009	
AUC comparison	Δ AUC	95%CI	SE	*P*-value*
Neuro Fellow ~ Resident	0.014	0.00-0.028	0.007	0.434
Resident ~ Rad Fellow	0.028	-0.011-0.068	0.02	0.158
Neuro Fellow ~ Rad Fellow	0.043	0.003-0.082	0.012	0.037

HET Density	AUC	95%CI	SE	
Neuroradiology Fellow	0.545	0.510-0.582	0.017	
Resident	0.542	0.505-0.579	0.015	
Radiology Fellow	0.539	0.502-0.575	0.017	
AUC comparison	Δ AUC	95%CI	SE	*P*-value*
Neuro Fellow ~ Resident	0.003	-0.022-0.028	0.013	0.814
Resident ~ Rad Fellow	0.003	-0.022-0.029	0.013	0.789
Neuro Fellow ~ Rad Fellow	0.007	-0.01-0.023	0.008	0.437

Swirl Sign	AUC	95%CI	SE
Neuroradiology Fellow	0.562	0.525-0.598	0.017

Resident	0.556	0.519-0.592	0.018	
Radiology Fellow	0.535	0.498-0.572	0.017	
AUC comparison	Δ AUC	95%CI	SE	*P*-value*
Neuro Fellow ~ Resident	0.006	-0.004-0.017	0.005	0.254
Resident ~ Rad Fellow	0.021	-0.021-0.063	0.022	0.332
Neuro Fellow ~ Rad Fellow	0.027	0.014-0.068	0.021	0.194

Black Hole Sign	AUC	95%CI	SE	
Neuroradiology Fellow	0.527	0.490-0.563	0.017	
Resident	0.512	0.476-0.549	0.011	
Radiology Fellow	0.503	0.466-0.540	0.001	
AUC comparison	Δ AUC	95%CI	SE	*P*-value*
Neuro Fellow ~ Resident	0.011	-0.002-0.023	0.006	0.095
Resident ~ Rad Fellow	0.013	-0.019-0.045	0.017	0.431
Neuro Fellow ~ Rad Fellow	0.024	-0.009-0.057	0.017	0.162

Blend Sign	AUC	95%CI	SE	
Neuroradiology Fellow	0.513	0.463-0.562	0.026	
Resident	0.511	0.460-0.564	0.026	
Radiology Fellow	0.502	0.451-0.554	0.026	
AUC comparison	Δ AUC	95%CI	SE	*P*-value*
Neuro Fellow ~ Resident	0.002	-0.017-0.021	0.010	0.815
Resident ~ Rad Fellow	0.009	-0.014-0.031	0.012	0.452
Neuro Fellow ~ Rad Fellow	0.011	-0.011-0.033	0.011	0.323

Fluid Sign	AUC	95%CI	SE	
Neuroradiology Fellow	0.505	0.468-0.542	0.012	
Resident	0.516	0.479-0.553	0.011	
Radiology Fellow	0.505	0.469-0.542	0.007	
AUC comparison	Δ AUC	95%CI	SE	*P*-value*
Neuro Fellow ~ Resident	0.001	-0.004-0.007	0.003	0.5817
Resident ~ Rad Fellow	0.003	-0.002-0.008	0.003	0.263
Neuro Fellow ~ Rad Fellow	0.004	0.000-0.009	0.002	0.040

Hypodensities	AUC	95%CI	SE	
Neuroradiology Fellow	0.565	0.528-0.608	0.019	
Resident	0.572	0.535-0.608	0.019	
Radiology Fellow	0.561	0.524-0.597	0.019	
AUC comparison	Δ AUC	95%CI	SE	*P*-value*
Neuro Fellow ~ Resident	0.001	-0.014-0.028	0.011	0.505
Resident ~ Rad Fellow	0.011	-0.01-0.031	0.010	0.289
Neuro Fellow ~ Rad Fellow	0.004	-0.017-0.025	0.011	0.708

* *P*-value of the test of DeLong et al. (1988)

Legend: Receiver operating characteristic curves for Noncontrast Computed Tomography Markers (NCCT) in the prediction of revised hematoma expansion (above row) and pairwise comparison of ROC analysis (below row) with difference in area under the curve (Δ AUC) across two Resident s and one neuroradiology fellow with extensive stroke imaging experience. AUC indicates area under the curve; 95% CI, confidence interval; HET density, heterogeneous density; IRR shape, irregular shape; Neuro Fellow, neuroradiology fellow; Rad Fellow, radiology fellow; SE, standard error.
